# Development of films based on chitosan, gelatin and collagen extracted from bocachico scales (*Prochilodus magdalenae*)

**DOI:** 10.1016/j.heliyon.2024.e25194

**Published:** 2024-01-24

**Authors:** María A. Moreno-Ricardo, Paula Gómez-Contreras, Ángel Darío González-Delgado, Joaquín Hernández-Fernández, Rodrigo Ortega-Toro

**Affiliations:** a Food Packaging and Shelf Life Research Group (FP&SL), Food Engineering Department, Universidad de Cartagena, Cartagena de Indias, 130001, Colombia; b Nanomaterials and Computer-Aided Process Engineering Research Group (NIPAC), Chemical Engineering Department, Universidad de Cartagena, Avenida del Consulado St. 30, Cartagena de Indias, 130015, Colombia; c Chemistry Program, Department of Natural and Exact Sciences, San Pablo Campus, University of Cartagena, Cartagena, 130015, Colombia; d Chemical Engineering Program, School of Engineering, Universidad Tecnológica de Bolivar, Parque Industrial y Tecnológico Carlos Vélez Pombo, Km 1 Vía Turbaco, Turbaco, 130001, Colombia; e Department of Natural and Exact Science, Universidad de la Costa, Barranquilla, 30300, Colombia

**Keywords:** Biocompatibility, Compression molding, Hydrophobic, Rigidity, Citric acid

## Abstract

Biodegradable biopolymers from species of the animal kingdom or their byproducts are sustainable as ecological materials due to their abundant supply and compatibility with the environment. The research aims to obtain a biodegradable active material from chitosan, gelatin, and collagen from bocachico scales (Prochilodus magdalenae). Regarding the methodology, films were developed from gelatin, chitosan, and collagen from bocachico scales (Prochilodus magdalenae) at different concentrations using glycerol as a plasticizer and citric acid as a cross-linker. The films were obtained with the hydrated mass processed by compression molding and characterized according to humidity, water solubility, contact angle, mechanical properties, and structural properties. The results of the films showed a hydrophobic characteristic. First, the chitosan-collagen (CS/CO) films showed a yellowish color, while the gelatin-collagen (Gel/CO) films were transparent and less soluble than the gelatin-collagen (Gel/CO) films. Concerning mechanical properties, gelatin films showed higher stiffness and tensile strength than chitosan films. Furthermore, in the morphological analysis, more homogeneous chitosan films were obtained by increasing the concentration of citric acid. In general, chitosan, gelatin, and collagen extracted from the scales of the bocachico (Prochilodus magdalenae) are an alternative in the application of films in the food industry.

## 1 Introduction

Currently, the development of sustainable alternative materials is necessary to combat environmental pollution and the depletion of natural resources caused by non-renewable petroleum-derived plastic materials due to the increasing demand for food and food packaging. Due to the growth of the world population and, in turn, the demand by consumers for safer alternatives is constantly increasing.

Many materials, such as plastic, paper, cardboard, steel, and aluminum, find extensive use across the global food industry. Synthetic plastics are prominent because of their affordability, lightweight nature, superior performance, and exceptional mechanical durability [1]. However, they represent a problem today because of their non-biodegradable and non-recyclable characteristics, contributing to significant environmental apprehension caused by the accumulation of considerable waste. Recent research indicates that around 85 % of plastic waste is eventually deposited in landfills and oceans, presenting a critical menace to soil quality, marine ecosystems, and biodiversity [2]. Therefore, substituting traditional materials with biodegradable alternatives, like biopolymers, holds the potential for sustainability and offers opportunity to implement the goals set forth by the international community, such as the Sustainable Development Goals (SDGs) established by the United Nations [3]. These goals provide a comprehensive framework for addressing pressing environmental concerns and promoting sustainable development worldwide. In this context, biodegradable biopolymers derived from animal kingdom, species or their residues offer a promising avenue for developing environmentally friendly materials. Their abundance and biocompatibility make them sustainable alternatives that align with the SDGs' objectives, particularly those related to responsible consumption and production, biodiversity conservation, and mitigating climate change [4]. By embracing these biopolymers within legal frameworks, it is possible to actively contribute to achieving the sustainable future envisioned by the United Nations and pave the way for a greener and more resilient planet.

Indeed, thanks to their impressive characteristics, such as superior gas barrier capabilities, mechanical strength, and film-forming properties, numerous bio-based polymers can be viable alternatives to conventional plastics [5]. Biobased polymers, such as polysaccharides, proteins, or combinations of these, such as chitosan, gelatin, and collagen, have been used [6,7]. Chitosan results from the deacetylation of chitin and gelatin from the partial hydrolysis of collagen. Both substances are biodegradable, compatible with biological systems, and devoid of toxicity [8]. Chitosan is widely used in the production of biodegradable materials due to its excellent film-forming properties, antimicrobial properties, and the capacity to retain bioactive substances and slowly release them within the realm of edibles [9,10]. On the other hand, gelatin is soluble in water and is a good biopolymer for forming transparent and flexible films [11]. According to studies, chitosan can prevent microbial deterioration in food due to the attraction between the positively charged amine groups in chitosan and the negatively charged charges on the microbial surface. This interaction causes the release of cellular components and eventual cell death, allowing the application in films and coatings, as it can preserve food quality and extend shelf life [12].

Even though films produced using chitosan or gelatin display favorable gas barrier characteristics, they come with certain limitations, like inadequate mechanical attributes in gelatin films and subpar water resistance in films made from gelatin and chitosan, restricting its potential application in packaging for food products. Different researchers have reported various approaches to enhance the characteristics of films based on chitosan and gelatin, including methods like cross-linking or blending with other polymers, such as polysaccharides, proteins, and lipids, alongside the inclusion of fillers and other additives [11,13].

On the other hand, collagen stands as the predominant protein in vertebrates and constitutes about 25 % of the total proteins of vertebrates [14]; This protein is characterized by its fibrous structure and ability to break down in an environmentally friendly manner naturally and film formation capacity [15]. It comprises three alpha-polypeptide chains arranged in a triple helix structure, running parallel. These chains associate with each other to create organized reticulate fibrils. These fibrils further combine to create solid fibers. Thanks to the triple solid helix configuration, these fibers play a crucial role in giving the extracellular matrix its durability and ability to withstand mechanical forces, for which the mechanical properties of collagen films present high resistance. Mechanically being stronger than edible polysaccharide films [16]. Thought to its unique triple helix structure, the approximate pH at which collagen reaches its isoelectric point is 7, which results in more waterproof collagen-based films. According to investigations, collagen-based films have high mechanical resistance, that is to say, that they are capable of resisting external pressure well, in order to ensure the integrity of what they contain [17]; while in gelatin films the amino acids present in the protein structure allow it to easily absorb UV radiation, protect food products of oxidative damage, in addition, gelatin includes in its functional properties, water retention capacity, formation capacity of films, helping in the formation of a flexible film, so promoting gelatin and collagen as viable alternatives for creating biodegradable packaging films [18].

Therefore, in this work the use of scales was considered as a by-product generated in the commercialization of the bocachico (*Prochilodus magdalenae*) to obtain collagen, being this fish is the most commercially important freshwater species in Colombia [19]; in this way added value is given to the scales and at the same time the properties that constitute it are used, which generates a new alternative in the development of food packaging materials. In this context, the objective of this work was to generate value for the scales in the sale of bocachico through the extraction of the collagen present in this by-product and creating composite films by combining chitosan/collagen and gelatin/collagen by compression molding with improved properties. This study can apply the groundwork for using composite films made from gelatin/collagen and collagen/chitosan in food preservation applications.

## 2 Materials and methods

### 2.1 Materials

Sigma Aldrich supplied the chitosan with reduced molecular weight (C.S.) (Bogotá, Colombia), food grade gelatin (Gel) (180 Bloom) was purchased from Districondorito (Cartagena, Colombia), Citric acid (C.A.), utilized as a crosslinker, and glycerol employed as a plasticizer, and acetic acid, ethylenediaminetetraacetic acid (EDTA), odium hydroxide (NaOH), and sodium hypochlorite (NaClO) were provided by Elementos Químicos Ltda (Bogotá, Colombia). Collagen (C.O.) was obtained from bocachico scales.

### 2.2 Extraction of collagen from bocachico (*Prochilodus magdalenae*) scales

Bocachico scales (*Prochilodus magdalenae*) supplied by the Lion City S.A.S. fish market (Cartagena, Colombia) with less than 24 h of slaughtering were used, which were transported to the experimental facilities of the Food Engineering program of the University of Cartagena. The preliminary treatments and collagen extraction were conducted following the methodology proposed through with some modifications [20]. In extracting collagen from the tilapia skin, a complete removal of the organic material adhered to it was initially carried out; for its part, the scale was dried and processed. In this process, all the substances employed were of analytical quality. The scales were washed with sodium hypochlorite (NaClO) at 250 ppm, followed by washing with distilled water to eliminate possible traces of NaClO. In order to eliminate proteins other than collagen present in the scales that can become contaminants at the end of the process, basic hydrolysis was carried out exposed to 0.1 N sodium hydroxide (NaOH) for a 24-h period, changing the solution every 12 h using a ratio of 1 part sample/solution to 10 parts (w/v) with constant agitation using a mechanical shaker at 450 rpm, the ratio and agitation were kept constant during all the treatments carried out; subsequently, the scales were washed with distilled water until neutral pH was obtained in the washing water. After the basic hydrolysis, the scales were decalcified due to their high calcium content, which would significantly affect the purity of the collagen. A 0.5 M ethylenediaminetetraacetic acid (EDTA) solution was used, adjusting the pH to 7.5. with sodium hydroxide (NaOH) for 48 h with gentle agitation, changing the solution every 24 h; the decalcified scales were washed with distilled water to remove traces of the reagents used. Next, to solubilize and recover the collagen in the scales in an acidic medium, a 0.5 M acetic acid (CH₃-COOH) solution was used for 48 h, followed by a filtration process to remove undissolved solids. In the acetic acid solution, a 12 % sodium chloride (NaCl) solution was added to the filtered solution to precipitate the collagen, thanks to the ionic charges that this salt provides to the protein, allowing its precipitation. Finally, a new filtration was carried out with a 20-mesh cloth, allowing the collagen to be recovered.

### 2.3 Preparation of the mixtures and obtaining of the films

For the preparation of the monolayer collagen and chitosan films, collagen extracted from tilapia skins, chitosan and glycerol were used according to the different formulations established in Table 1 and gelatin, chitosan, gelatin/collagen (Gel/CO), chitosan/collagen (CS/CO) -based films were prepared by compression molding. The chitosan hydrated mass (C.S.) was prepared according to the methodology proposed by Guerrero (2019) [21], with some modifications determined by preliminary tests. Table 1 presents the formulations, following a straightforward classification design featuring two control groups (pure C.S. and pure Gel) was used to obtain a total of 6 formulations. The levels were adjusted with preliminary trials. The production of the monolayer composed of collagen and chitosan was carried out through the casting process due to the restrictions imposed by the thermal properties of collagen, which prevented its processing in compression molding without experiencing thermal degradation. The chitosan was mixed with citric acid powder at 20 % (w/w, using chitosan as the base) since this percentage of citric acid yielded the most favorable outcome in forming chitosan films. Subsequently, distilled water was added in a 1:3 (w/v) chitosan/water ratio to the powder mixture to facilitate the flow of chitosan during the compression process. Finally, 15 % glycerol was added dropwise (p/p, based on chitosan). In this sense, a compact wet dough was formed with the appropriate properties for compression molding. The film-forming solution was dried at 45–50 °C in 20 cm × 20cm Teflon trays and conditioned for seven days in desiccators with a supersaturated sodium bromide (56 % RH) until characterization.

**Table 1:** Experimental design to obtain biodegradable films (mass fraction).

Formulations	Gelatin	Chitosan	Collagen	Glycerol	Citri acid
F1	0.87	0.00	0.00	0.13	0.00
F2	0.00	0.74	0.00	0.11	0.15
F3	0.79	0.00	0.08	0.13	0.00
F4	0.67	0.00	0.20	0.13	0.00
F5	0.00	0.68	0.07	0.11	0.14
F6	0.00	0.59	0.18	0.12	0.12

Regarding the hydrated mass of gelatin, the methodology used in the chitosan was modified, adjusting it according to preliminary tests to the gelatin processing conditions, distilled water was mixed in a proportion of 2 parts water to 1 part gelatin (w/v), and 15 % dropwise glycerol (w/w, based on gelatin). The formulations containing collagen were added at the beginning of the preparation of the dough, in which the materials were mixed manually. The masses were placed in a polymer bag and kept at room temperature for 24 h.

The films were obtained with the hydrated mass processed by compression molding. For which a press was used, previously heating it to 55 °C to obtain gelatin-based films and to 125 °C for chitosan films (so that the polymer blends reach their softening temperature, and the molding process is efficient); the blend was positioned between the two pressing plates, allowed to heat for 1 min without applying pressure, and then pressed at 2.5 MPa for 2 min. Finally, the pressure was released, and the films were de-molded. The films were conditioned in a desiccator with sodium bromide until their analysis at a relative humidity of 58 % and a temperature of 25 °C.

### 2.4 Characterization of the material

#### 2.4.1 Color

The surface color of the films was measured using a portable colorimeter (Colorimeter CHN Spec CS-10) using the regular lighting conditions of D65, an observing angle of 10°, and calibrated based on the standard black and white color using the CIELab system. The CIELab scale was employed for color parameter measurement: L* ranging from 0 (black) to 100 (white), -a* representing greenish hues to +a* for red tones, and -b* indicating blue shades to + b* denoting a high presence of yellow color. The h* value represented the overall chromatic difference, while C* indicated the intensity. The films were positioned on a white standard with color parameters: L* at 97.53, a* at 0.04, and b* at 2.07. Each sample underwent five measurements. The value of the total chromatic difference (ΔE) was calculated using equation (1)

(1) $$\Delta E=\sqrt{{\left(L_{0}^{*}+L_{K}^{*}\right)}^{2}+{\left(a_{0}^{*}+a_{k}^{*}\right)}^{2}+{\left(b_{0}^{*}+b_{k}^{*}\right)}^{2}}$$

The subscript 0 represents the control film, and k is the collagen sample.

#### 2.4.2 Glow

Glossiness was assessed at a 60° angle of incidence, following the ASTM D523 standard [22] standard procedure, employing a gloss meter with a flat surface (3nh Gloss Meter). Three films were analyzed for every combination, with three readings taken from each sample. The outcomes are presented in gloss units (GU), compared to a precisely polished standard of polished black glass having a gloss importance of 96 GU.

#### 2.4.3 Thickness

The thickness was measured with the use of a digital micrometer. Eight measurements were randomly collected from the film, and the mean value and corresponding standard deviation were subsequently documented. These measurements were taken in triplicate for more excellent reliability of the values.

#### 2.4.4 Moisture content

The films were dried in a natural convection muffle at 65 °C for 12 h, and continued heating was carried out to achieve a consistent weight. The humidity percentage was computed by dividing the weight of the film when wet by its weight when dry. The mean values were calculated as the average of three repetitions for each formulation.

#### 2.4.5 Water solubility

The films were placed into distilled water using film a ratio of 1 part water to 10 parts (w/v) for 48 h. Subsequently, the samples underwent a 24-h drying in a natural convection oven at 65 °C to eliminate free water. Next, they were situated in a desiccator containing a saturated sodium bromide (NaBr) solution (RH = 58 %, T = 25 °C) for two weeks to eliminate any bound water present. Finally, the solubility percentage of the films was determined based on the starting and eventual weights. This procedure was replicated three times for each formulation, and the calculated arithmetic mean was reported.

#### 2.4.6 Contact angle

The determination was made by observing the form of a 0.01 ml water droplet after 20 s. A sample of the film (1 cm^2^) was placed on a horizontal surface with a white background, and a drop of water was placed on the film's surface. The image was captured using a digital camera; the distance to the water droplet in the space that separates the camera eyeglasses remained consistent at a parameter of 50 cm. The reported image was analyzed using the Goniotrans Software. The process was performed three times for each formulation, reporting the arithmetic average.

#### 2.4.7 Water vapor permeability

The determination was carried out using a gravimetric approach, adhering to the established ASTM E96-95 procedure [23] with certain adjustments. Changes were made to create a humidity gradient from 58 % RH to 100 % at 25 °C. Films without physical defects were chosen for Water Vapor Permeability (WVP) testing. Payne permeation cups containing distilled water were used, exposing one side of the film to 100 % RH. These cups and the films were placed in humidity-controlled cabinets at 25 °C. The cabinet RH (58 %) was maintained using supersaturated sodium bromide solutions. Also, to act the practical use of the films on products with high water activity, the free surface of the film during its creation was exposed to lower relative humidity. The glasses carrying the films were periodically weighed using a precision analytical balance (0.0001 g). Once the measurements reached a stable state, the water vapor transmission rate (WVTR) was calculated by analyzing the slope of the regression curve, plotting weight loss against time. This value was then divided by the film's area. The entire test was conducted three times, and the reported results include the average value and the corresponding standard deviation.

#### 2.4.8 Mechanical properties

The procedure was carried out according to what was stated in the ASTM D882 [24], standard. Essential characteristics such as elastic modulus (EM), tensile strength (TS), and deformation (E) of the examined films were assessed. The equipment employed for the investigation comprised a TA.XT plus model universal testing machine (Stable et al.) equipped with a 500 N load cell and operated at a head speed of 50 mm/min. The film specimens had dimensions of 25 mm × 100 mm, and the distance between the clamps was set at 50 mm. The measurement of the thickness of the different samples was carried out by taking five measurements with the use of a digital micrometer. Before determining each of these properties, the samples to be analyzed were cut and conditioned in a relative humidity of 58 % at a temperature of 25 °C for 48 h. The mean value and the corresponding standard deviation were documented.

#### 2.4.9 Scanning electron microscopy (SEM)

Microscopic examinations were performed using a Hitachi model SU8010 scanning electron microscope. The films underwent vacuum drying, fracture analysis, and freezing with liquid nitrogen, and coated with an Au–Pd layer. These prepared samples were attached to copper slides and observed using an accelerating voltage of 6 kV.

### 2.5 Statistical analysis

The data were analyzed employing Statgraphics Plus for Windows 5.1 (Manugistics et al. MD). An assessment of variance using (ANOVA) was conducted, and the averages were compared using Fisher's Least Significant Differences (LSD) test, with a 95 % certainty level.

### 2.6 Application of fish collagen as a coating for cape gooseberries with gelatin and chitosan

Film-forming solutions were prepared from fish collagen, gelatin, and chitosan. Subsequently, food samples were packaged at refrigerated (10 °C) and ambient (30 °C) temperatures and monitored for weight, color, texture, and appearance. The results were evaluated using Statgraphics Plus for Windows 5.1 software (Manugistics Corp., Rockville, MD), and an analysis of variance (ANOVA) was performed. The means were then compared using Fisher's Least Significant Differences (LSD) test with a 95 % confidence level.

## 3 Results and discussion

### 3.1 Optical properties of the film

The color and gloss of the surface of the films based on gelatin, chitosan, and collagen as additives were studied, and the findings are presented in Table 2. The appearance of the color of the films directly connects to the product's visibility and the consumers' acceptance [25], and gloss is associated with the surface morphology of the films [26].

**Table 2:** Mean values and standard deviation of brightness (gu) and color parameters (luminosity (l^a^), red/green (a^a^), yellow/blue (b^a^), chromaticity (c^a^) and hue angle (h°)).

Formulation	Brightness (GU)	Color parameters					
		L^a^	a^a^	b^a^	h°	C^a^	ΔE
F1	28.75 ± 0.46^c^	89.05 ± 0.49^b^	1.24 ± 0.28^a^	12.21 ± 0.39^a^	12.28 ± 0.4^c^	84.21 ± 1.18^ab^	–
F2	3.75 ± 0.71^a^	74.06 ± 3.83^a^	2.5 ± 0.47^ab^	34.1 ± 2.73^b^	34.21 ± 2.81^d^	85.93 ± 2.25^b^	–
F3	21.5 ± 1.77^b^	89.92 ± 0.86^b^	1.29 ± 0.22^a^	8.98 ± 0.55^a^	9.07 ± 0.52^a^	81.73 ± 1.76^a^	3.35^a^^a^
F4	20.13 ± 1.55^b^	89.48 ± 0.38^b^	4.61 ± 0.79^b^	10.87 ± 0.48^a^	10.94 ± 0.48^b^	83.28 ± 0.24^a^	3.66^a^^a^
F5	3.63 ± 0.52^a^	76.76 ± 1.53^a^	1.76 ± 0.75^a^	29.46 ± 4.72^b^	29.54 ± 4.83^d^	86.87 ± 2.65^b^	5.41^b^^b^
F6	3.38 ± 0.52^a^	76.18 ± 1.25^a^	2.06 ± 0.66^ab^	28.95 ± 3.07^b^	29.03 ± 3.11^d^	85.97 ± 0.88^b^	5.58^b^^b^

Different letters within the same column indicate significant differences between the formulations (p < 0.05).

a Parameter compared to F1.

b Parameter compared to F2.

The color characteristics of the films are presented in Table 1. The L* value indicates the film's brightness, while the* value indicates the film's degree of redness or greenness, collagen showed no significant impact on the L* values in the different films; the b* values signify the yellow or blue color level in the film, with higher b values indicating a yellower appearance, as observed in the chitosan-collagen (CS/CO) films. The ΔE value accounts for the overall color distinction of the film sample. An ΔE value exceeding 5 indicates a noticeable difference in the film's color [27].

For the gelatin-collagen films (Gel/CO), these proved to be color less, since their value is less than 5. According to how collagen was added in the chitosan-collagen films (CS/CO) matrix, films had varying degrees of yellowness, increasing significantly from 3.35 to 3.66. The L* value of the films (Gel/CO) decreased, however, the brightness of the films increased.

### 3.2 Physical properties of films

The behavior of the films about water was studied, calculating thickness, contact angle (CA), moisture content (MC), water solubility (WS), and water vapor permeability (WVP) reflected in Table 3.

**Table 3:** Mean values and standard deviation of thickness, contact angle (CA), moisture content (MC), water solubility (SA) and water vapor permeability (WVP) of the studied formulations.

Formulation	Thickness (μm)	CA (°)	MC (%)	WS (%)	WVP (g*mm/kPa*h*m^2^)
F1	277.38 ± 11.39^b^	79.4 ± 2.07^a^	11.63 ± 0.42^bc^	44.04 ± 2.70^d^	2.96 ± 0.03^b^
F2	345.75 ± 10.90^d^	80.8 ± 2.39^a^	12.62 ± 0.18^c^	16.81 ± 0.98^b^	3.37 ± 0.06^c^
F3	233.50 ± 11.06^a^	81.8 ± 1.3^a^	10.72 ± 0.68^b^	43.61 ± 1.12^d^	2.97 ± 0.86^b^
F4	224.38 ± 10.58^a^	84.6 ± 1.14^b^	8.42 ± 0.8^a^	40.02 ± 0.65^c^	3.12 ± 0.47^bc^
F5	285.25 ± 24.88^c^	83.8 ± 1.92^ab^	12.09 ± 0.3^c^	13.75 ± 1.70^a^	2.50 ± 0.09^a^
F6	276.50 ± 23.83^c^	85.6 ± 1.34^b^	10.95 ± 0.51^b^	19.08 ± 1.76^b^	3.30 ± 0.81^bc^

Different letters within the same column indicate significant differences between the formulations (p < 0.05).

The films showed a surface of a hydrophobic nature since its angles were in the ranges of 79–85°. The results are similar to biopolymer films based on chitosan and collagen, which showed values between 79 and 103°, considering that the increase in degree° is due to the creation of hydrogen bonds between chitosan's hydroxyl groups and collagen's amino groups. Similarly, the hydrophobic characteristic of chitosan causes less interaction with water, which explains why the film's surface results in a reduced hydrophilic group and causes an increase in the hydrophobicity of the films [28].

### 3.3 Mechanical properties

The mechanical properties are related to structure and intermolecular interactions within the film matrix. The values obtained for Tensile Strength (TS), extension at the point of rupture or fracture point (EB), and modulus of elasticity or Young's modulus (E) are presented in Table 4.

**Table 4:** Mean values and standard deviation of the tensile strength (TS), maximum strain at the fracture point (E) and elastic modulus (em) of the studied films.

Formulation	TS (Mpa)	E (%)	EM (Mpa)
F1	68 ± 2^c^	12.1 ± 0.8^a^	1700 ± 8^b^
F2	41 ± 2^a^	20.2 ± 0.5^b^	690 ± 5^a^
F3	71 ± 3^d^	11.9 ± 0.2^a^	1720 ± 10^c^
F4	76 ± 2^d^	11.5 ± 0.4^a^	1780 ± 9^d^
F5	50 ± 2^b^	21 ± 0.5^b^	680 ± 11^a^
F6	55 ± 3^b^	20.6 ± 0.2^b^	695 ± 9^a^

Different letters between the columns mean significant differences between the formulations (p < 0.05).

Examining the mechanical characteristics (Table 4) indicates that in gelatin films, the TS increases significantly with the addition of collagen. The rigidity has an increasing tendency in gelatin films, and in chitosan films, they do not present significant differences (p < 0.05). In chitosan films, the addition of collagen presents a similar behavior since the addition of collagen increases the values. This increase is due to collagen since it quickly absorbs water. Therefore, the gelatin film has a higher resistance than the chitosan film because the higher the tensile stress value, the higher the film's resistance due to the polymers ' interaction [29].

Edible chitosan-gelatin films report that the chitosan film had a significantly lower tensile strength (18.252 MPa) but a more excellent elongation at break (39.821 %) than gelatin and composite films [30]. Some authors also found that acids could act as plasticizers in the film, which decreased the tensile strength and significantly increased the elongation at break [31,32].

### 3.4 Morphological analysis

**Fig. 1:**
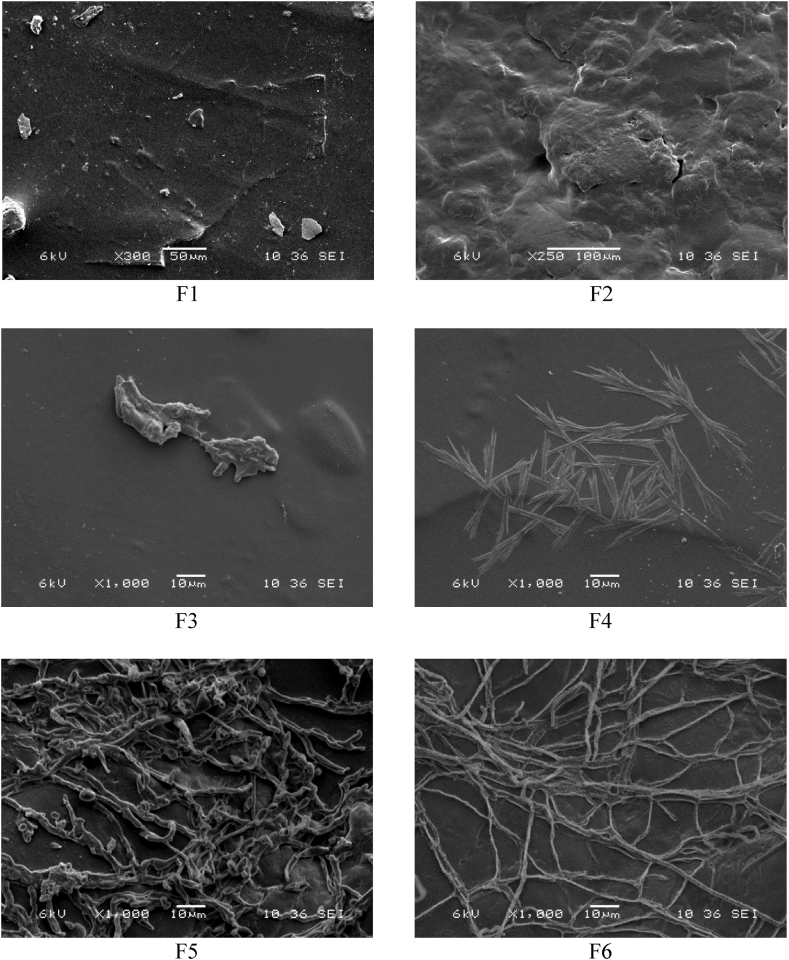
Surface scanning electron micrographs of the films studied (F1–F6 means the biodegradable film formulations respectively).

SEM analyzed the surface morphology of the films to examine how the inclusion of collagen affects the composition of the gelatin and chitosan control films. The SEM images are shown in Fig. 1.

Fig. 1 (F1), which represents the surface morphology of the gelatin film, shows a compact film with a flat and smooth appearance with an ordered and homogeneous structure (without pores or cracks. In Fig. 1 (F2) no phase separation was observed, repressenting a solid compatibility among chitosan, citric acid, and glycerol. In Fig. 1 (F3–F6), this miscibility in a mixture of polymers results from distinct interactions among the polymer constituents. The presence of cracks in the surface morphology of chitosan films can be attributed to the inherent low flowability of solid chitosan [21]. The glycerol in the chitosan films favors the fluidity of the chitosan chains. In turn, the process of chitosan crystallization does not alter the inherent chitosan structure, unlike citric acid, as a crosslinking agent, modifies the chitosan structure, giving rise to an amorphous material [21]. It was observed in the preliminary tests of the investigation that as the concentration of citric acid increased, more homogeneous chitosan films were obtained.

Similar studies indicate that it is difficult to identify gelatin and collagen in composite films. Consequently, gelatin could be evenly distributed in the composite films and closely combined with collagen fibers to form a hierarchical structure [33,34]. In films with different proportions of chitosan/gelatin, they have shown a homogeneous structure without apparent phase separation, indicating excellent compatibility and strong interactions between the two biopolymers, especially when the addition of gelatin to chitosan makes the films brighter, more transparent, and amorphous [35].

### 3.5 Effect of coating on the conservation of cape gooseberries

**Fig. 2:**
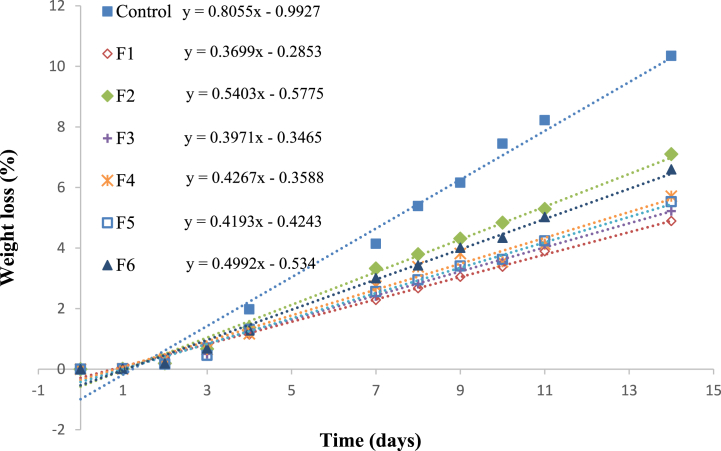
Weight loss of cape gooseberries without and with coating of all the formulations studied, stored at room temperature (25 °C) for two weeks.

Freshness is essential to evaluate the application of coating films on fruits, since, the rate of water loss that occurs through metabolic processes such as transpiration and respiration of the fruit after the postharvest stage can be determined [36,37]. In this way, Fig. 2 shows the properties of cape gooseberries with and without coating, stored at room temperature (25 °C) for 15 days. The uncoated cape gooseberries experienced accelerated weight loss after 15 days of storage, and from day 4, they showed a high deterioration rate, presenting softening in their rind.

On the other hand, the coated cape gooseberries showed a slower deterioration, so their deterioration began to be noticed after day 8. In addition, it was observed that, when applying these materials as a coating on cape gooseberry, the weight loss was delayed by 50 % the percentage of weight loss due to dehydration, this was reflected in F3, compared to F1. Similar studies developed a film from collagen from discarded fish skin, carboxymethyl cellulose (CMC) as a cross-linker, and Berberis lyceum root extract (BLRE) as an antioxidant agent and evaluated its shelf life with mushrooms, in which they observed changes in color and wrinkling of the surface, as a result of loss of moisture and cloudiness of the cells. On the first day of canning, all the mushrooms were white and had a smooth surface. After seven days, the unpackaged mushroom shows a dry, dull surface with fungal growth noticeable on the surface), and its color changes to black, showing significant deterioration. In this way, mushrooms packaged in CCMC +2 % BLRE and CCMC +4 % BLRE films have high moisture content, are less wrinkled, and change color less [38].

Likewise, one study developed a film based on collagen, with the addition of an antimicrobial agent (nisin), to analyze the reduction of water loss and fat oxidation caused by microorganisms, applied to fillets of pork preserved in refrigeration for ten days, the results indicate that the meat samples that were not covered by the collagen films exhibited a high percentage of moisture loss (34.57 %) different from those that were covered by the films, managing to reduce moisture loss in pork fillets by up to 11.87 % [39].

Importantly, evaporation at the top of the product is a process that absorbs heat, leading to a decrease in product temperature. The amount of water lost during this process is influenced by internal characteristics, such as surface shape, size, surface area to weight/volume ratio, state of maturity, and possible physical damage. In addition, it is also influenced by external factors such as temperature, relative humidity, and air circulation around the product [36]. On the other hand, using chitosan in coating increases weight loss, firmness, and antioxidant activity, as well as the content of soluble solids in fresh fruits and vegetables [40].

## 4 Conclusions

In this study, chitosan, gelatin, and collagen extracted from Bocachico Scales (Prochilodus magdalenae) are presented as an ecological resource that can be used to produce multifunctional, recyclable, biocompatible, and biodegradable materials in the production of films. However, it is necessary to continue exploring the biodegradability of films in different environments. In general, all films reflected a hydrophobic nature. The gelatin/collagen (Gel/CO) films showed good properties about water and appeared to be transparent. In contrast, the chitosan/collagen (CS/CO) films showed excellent mechanical properties with a yellowish color. Besides, gelatin films show high values, making them more rigid than chitosan films. In the same way, it is observed in the morphological analysis that the greater the amount of collagen added in chitosan films, the more collagen fiber networks are observed, which may be related to the increase in tension when this protein is added, resulting in a strong film. However, rigidity and elongation are unaffected since the collagen fibers are not fully integrated into the surface. In general, this study demonstrates a new alternative for developing collagen-based films, which is significant in applying films in the food industry with ecological and economical alternatives to conventional plastic films. This offers a promising contribution to addressing environmental challenges and promoting responsible consumption and production, as biodegradable polymers demonstrate potential in replacing conventional plastics, reducing pollution, and mitigating the harmful impacts on ecosystems, supporting the transition towards a circular economy, where resources are efficiently utilized, and waste is minimized. By adopting sustainable practices, innovative research, and supportive legal frameworks, biodegradable polymers can contribute significantly to achieving the standards and goals set forth at the international level, such as the SDGs mentioned above, leading us towards a more sustainable and resilient future. Also, this type of fishing waste is relevant worldwide and even more so in South America, where these are usually thrown into garbage dumps where they cause pollution. Obtaining collagen from different types of waste is relevant since collagen is a macromolecule with high added value with applications in the food, pharmaceutical, cosmetic, and medical industries. With this type of film, materials can be developed for food packaging, facial masks for beauty, drug packaging, and wound patches where drugs are released in a controlled manner, among others, industries, and applications in which the products have even greater added value than in food packaging.

## Funding

Universidad de Cartagena, Colombia financially supported this research under project number 115–2019.

## Additional information

There is no additional information available for this document.

## CRediT authorship contribution statement

**María A. Moreno-Ricardo:** Methodology, Investigation. **Paula Gómez-Contreras:** Writing – review & editing, Writing – original draft, Investigation. **Ángel Darío González-Delgado:** Software, Project administration, Investigation, Funding acquisition. **Joaquín Hernández-Fernández:** Visualization, Investigation, Funding acquisition, Conceptualization. **Rodrigo Ortega-Toro:** Writing – review & editing, Investigation, Funding acquisition, Formal analysis, Conceptualization.

## Declaration of competing interest

The authors declare the following financial interests/personal relationships which may be considered as potential competing interestsPaula Gomez-Contreras reports financial support was provided by 10.13039/501100005741University of Cartagena.
